# Identification and complete genome sequencing of paramyxoviruses in mallard ducks (*Anas platyrhynchos*) using random access amplification and next generation sequencing technologies

**DOI:** 10.1186/1743-422X-8-463

**Published:** 2011-10-06

**Authors:** Toon Rosseel, Bénédicte Lambrecht, Frank Vandenbussche, Thierry van den Berg, Steven Van Borm

**Affiliations:** 1 Operational Directorate of Virology, Veterinary and Agrochemical Research Center, Ukkel, Belgium

**Keywords:** APMV4, APMV6, avian paramyxovirus, mallard, next generation sequencing, random amplification, SISPA

## Abstract

**Background:**

During a wildlife screening program for avian influenza A viruses (AIV) and avian paramyxoviruses (APMV) in Belgium, we isolated two hemagglutinating agents from pools of cloacal swabs of wild mallards (*Anas platyrhynchos*) caught in a single sampling site at two different times. AIV and APMV1 were excluded using hemagglutination inhibition (HI) testing and specific real-time RT-PCR tests.

**Methods:**

To refine the virological identification of APMV2-10 realized by HI subtyping tests and in lack of validated molecular tests for APMV2-10, random access amplification was used in combination with next generation sequencing for the sequence independent identification of the viruses and the determination of their genomes.

**Results:**

Three different APMVs were identified. From one pooled sample, the complete genome sequence (15054 nucleotides) of an APMV4 was assembled from the random sequences. From the second pooled sample, the nearly complete genome sequence of an APMV6 (genome size of 16236 nucleotides) was determined, as well as a partial sequence for an APMV4. This APMV4 was closely related but not identical to the APMV4 isolated from the first sample. Although a cross-reactivity with other APMV subtypes did not allow formal identification, the HI subtyping revealed APMV4 and APMV6 in the respective pooled samples but failed to identify the co-infecting APMV4 in the APMV6 infected pool.

**Conclusions:**

These data further contribute to the knowledge about the genetic diversity within the serotypes APMV4 and 6, and confirm the limited sensitivity of the HI subtyping test. Moreover, this study demonstrates the value of a random access nucleic acid amplification method in combination with massive parallel sequencing. Using only a moderate and economical sequencing effort, the characterization and full genome sequencing of APMVs can be obtained, including the identification of viruses in mixed infections.

## Background

A large number of viruses of humans and animals are classified in the family *Paramyxoviridae *[1]. Their single stranded, unsegmented, RNA genomes of negative orientation vary in length from 13-19 kb and contain 6-10 genes encoding up to 12 different proteins [1]. Avian paramyxoviruses (APMV) are frequently isolated from domestic and wild birds throughout the world. Recently they are classified in the genus *Avulavirus *of the subfamily *Paramyxovirinae*, family *Paramyxoviridae *[2]. Ten serological types (APMV1-10) of APMVs are described so far based on hemagglutination inhibition (HI) and neuraminidase inhibition tests [3-5]. APMV1, including Newcastle disease virus (NDV, defined in [6]) is the most characterized among all APMV types because it can cause severe disease outbreaks in poultry. In contrast to the well-studied APMV1 or NDV, very little is known about the biological characteristics, pathogenicity, and diversity (both genetic and antigenic) of other APMV serotypes 2-10. APMV types 2, 3, 6 and 7 have been associated with disease in domestic poultry [7-13]. APMV6 viruses have been associated with mild respiratory disease and decreased egg production in turkeys [14]. APMV3 and APMV5 (Kunitachi virus) caused severe pulmonary disease in wild birds [15,16]. Other serotypes, including APMV4, 8, 9 and 10 have been isolated from ducks, waterfowls, and other wild birds with no clinical signs of disease [3,5,17-20]. APMV4 viruses have been isolated predominantly from feral birds of the order *Anseriformes *[21,22] and from commercial ducks and geese, presumably as a result of their direct contact with feral waterfowl [21,23,24]. Experimental infection of chickens with APMV4 and APMV6 showed mild respiratory pathology, suggestive of possible viral disease in poultry [8,25].

Molecular characterization through whole genome sequencing of APMV2-10 remains technically challenging because these viruses are poorly represented in public sequence databases, complicating the design of sequencing primers. Recent efforts to sequence whole genomes of representative strains for all serotypes (APMV2 [26], APMV3 [27,28], APMV4 [29,30], APMV5 [31], APMV6 [25,32], APMV7 [33], APMV8 [34], APMV9 [35], APMV10 [5]) have significantly contributed to our understanding of the *Avulavirus *genus genome organisation. However, further studies are needed to explore the diversity within the serotypes.

Random access sequencing using sequence independent single primer amplification was previously described for NDV genome sequencing [36], based on resource demanding sequencing of high number of cloned random amplicons to achieve completion of a genome. This protocol contains efficient steps to enrich viral nucleic acids and deplete contaminating and host sequences, including size selective filtration and extensive nuclease treatments [36,37]. It was also used for the molecular identification of an APMV in penguins [5] where existing protocols did not allow a starting point for primer walking. This resulted in the identification of a new serotype, APMV10.

Massive parallel sequencing technologies were developed to accommodate the need of higher sequencing capacity and lower costs per nucleotide for large genome sequencing projects [38]. One main advantage of these second generation sequencing technologies is the possibility to sequence DNA samples without any prior knowledge of the sequence, which is required for priming [38].

During a wildlife screening program for avian influenza A viruses (AIV) and APMVs, we isolated two hemagglutinating agents from two pools consisting of each four cloacal swabs of wild mallards. The birds were caught in a same location at two different times. AIV and APMV1 were excluded using HI testing and specific real-time RT-PCR tests. To refine HI test based identification of these viruses and in lack of validated molecular tests for APMV2-10, this study applied random access amplification in combination with next generation sequencing for the sequence independent identification of the viruses and the determination of their complete genome sequences.

## Results

### Identification and genome sequence of avian paramyxoviruses

Two pooled samples, consisting of each four swab samples from wild mallards, were positive for hemagglutinating agents without inducing mortality of embryonated chicken eggs. AIV and APMV1 could be excluded using specific real-time RT-PCR tests (data not shown) and HI tests using reference sera for AIV and APMV1. The HI assays with reference sera specific for APMV2-9 identified sample mallard/Belgium/15129/07 as APMV4 positive and sample mallard/Belgium/12245/07 as APMV6 positive. A cross reactivity with the APMV2 reference serum P/Robin/Hiddensee/57 was observed for both samples, but not with another APMV2 reference serum P/chicken/Yucaipa/Cal/56. The HI titers for the APMV3 and APMV7 reference sera showed for sample mallard/Belgium/15129/07 the borderline value of 16, still we considered this as nonspecific reactivity (Table 1).

**Table 1:** Hemagglutination inhibition tests (HI-titers) of pooled samples mallard/Belgium/15129/07 (07/15129) and mallard/Belgium/12245/07 (07/12245)

Antisera (used inactivated virus strain)	07/15129	07/12245
Polyclonal serum APMV1	< 4	< 4
Monoclonal serum 12B7 anti APMV1		< 4
Monoclonal serum 8C11 + 4D6 anti APMV1		< 4
Polyclonal serum APMV2 (P/robin/Hiddensee/15/75)	32	64
Polyclonal serum APMV2 (P/chicken/Yucaipa/Cal/56)	< 4	8
Polyclonal serum APMV3 (P/tk/1087/82)	16	4
Polyclonal serum APMV4 (P/duck/Hong Kong/D3/75)	128	< 4
Polyclonal serum APMV6 (P/duck/Hong Kong/199/77)	< 4	64
Polyclonal serum APMV7 (P/dove/TN/4/75)	16	8
Polyclonal serum APMV8 (P/goose/Del/1053/76)	< 4	4
Polyclonal serum APMV9 (P/duck/NY/22/78)	< 4	< 4
28 influenza A reference sera tested *	< 4	< 4

**Genetic identification**	APMV4	APMV6 + APMV4

* sera specifications available on request

Combining the advantages of random amplification and massive parallel sequencing, 5225 and 12310 sequence reads were produced from the library resulting respectively from sample mallard/Belgium/12245/07 and mallard/Belgium/15129/07. More than 95% of these reads were specific for APMVs, and host-derived or contaminating sequences were negligible.

Assembly of random generated sequences for sample mallard/Belgium/15129/07 produced a 15054 nucleotides (nt) contig representing the complete genome sequence of an APMV4. APMV4/mallard/Belgium/15129/07 (APMV4-BE15129) was assembled from 9767 sequence reads of raw data (APMV4/KR/YJ/06 [GenBank:EU877976] used as reference in the reference assembly). Assembly of 4715 sequences generated for sample mallard/Belgium/12245/07 produced a nearly complete (98.89%) APMV6 genome of length 16236 nt (APMV6/mallard/Belgium/12245/07; APMV6-BE12245). APMV6/Goose/FarEast/4440/2003 [GenBank:EF569970] was used as a reference sequence in this reference assembly. Surprisingly, APMV4 sequences were also identified in sample mallard/Belgium/12245/07. APMV4/KR/YJ/06 [GenBank:EU877976] was used as a reference and 21 sequences mapped to various regions (total of 2977 nt representing 19.75% of the APMV4 genome sequence). The APMV4 virus was named APMV4/mallard/Belgium/12245/07 (APMV4-BE12245). Unfortunately the original individual cloacal swabs were no longer available at the time of the genetic analysis, so we could not find out which of the four animals in the pool were infected and whether we were dealing with a mixed infection of one bird. The missing 1.11% of the APMV6 genome represents two small internal gaps and some nucleotides (24 nt at 5' and 37 nt at 3') at the genome termini. A low coverage at the genome termini was also observed for the fully sequenced APMV4 genome (41 terminal 5' nt and 42 terminal 3' nt, with depth ≤ 3×).

### Database accession numbers

The consensus sequences were submitted to GenBank under the following accession numbers: JN571485 (APMV4/mallard/Belgium/15129/07, complete genome), JN57148 (APMV6/mallard/Belgium/12245/07, nearly complete genome) and JN571487, JN571488, JN571489, JN571490 (APMV4/mallard/Belgium/12245/07, partial sequences of phosphoprotein, fusion protein, hemagglutinin-neuraminidase and large polymerase genes).

### Genomic features of APMV4/mallard/Belgium/15129/07

The virus has a genome length of 15054 nt as previously described for APMV4 viruses, consisting of six transcriptional units (Table 2) encoding from 3' to 5' the NP (nucleoprotein), P/V/W (phosphoprotein and additional proteins through RNA editing), M (matrix), F (fusion), HN (hemagglutinin-neuraminidase) and L (large polymerase) proteins. The 3' leader and 5' trailer sequences of the genome were respectively 55 nt and 17 nt in length. Gene start and gene end sequences were as previously described for APMV4 [30]. The NP protein encoded a 457 amino acids (aa) protein, as previously described for other APMV4. The P gene encodes a 393 aa phosphoprotein. A putative RNA editing site at genome position 2057-2065 (5'AAAGGGGGG-3') was identified, where insertion of one non-templated G residue would encode a 224 aa V protein. Alternatively, the insertion of two non-templated G residues would result in a putative W protein of 137 aa. The matrix gene open reading frame (ORF) encodes a 370 aa long matrix protein, unlike the 367 aa or 369 aa previously described for APMV4 genomes [29,30]. The lengths of the other proteins encoded by their ORF's are the same as previously described for APMV4 (F 566 aa; HN 569 aa; L 2211 aa). The fusion protein has a monobasic cleavage site (DIQPR↓F).

**Table 2:** APMV4/mallard/Belgium/15129/07 genome organization and characterization (genome size 15054 nt)

Gene	Genome position	5'UTR (nt) (incl. gene-start)	ORF(nt)	3'UTR (nt) (incl. gene-end)	Intergenic region (nt)	Deduced protein size (aa)
NP	56-1606	60	1374	117	9	457
P (V; W)*	1616-2979	46	1182 (675; 413)	136	34	393 (224; 137)
M	3014-4306	77	1113	103	16	370
F	4323-6210	71	1701	116	40	566
HN	6251-8161	78	1698	135	45	565
L	8207-15037	92	6636	103		2211

*putative RNA editing site at position 2057-2065 (5'-AAAGGGGGG-3')

### Genomic features of APMV6/mallard/Belgium/12245/07

The genome length of 16236 nt is consistent with that of "class I" of APMV6 [32], containing seven transcriptional units (Table 3) encoding from 3' to 5' the NP, P/V/W, M, F, SH (small hydrophobic protein), HN and L proteins. The F protein has a monobasic cleavage site, PEPR↓L. The 3' leader and 5' trailer sequences of the genome were respectively 55 and 54 nt in length. Gene start and gene end sequences were as previously described for APMV6 [32]. The lengths of the proteins encoded by the ORF's are the same as previously described for APMV6 (NP 465 aa; P 430 aa, V 268 aa, W 177 aa, M 366 aa, F 555 aa, 142 aa, HN 613 aa, L 2241 aa).

**Table 3:** APMV6/mallard/Belgium/12245/07 genome organization and characterization (genome size 16236 nt)

Gene	Genome position**	5'UTR (nt) (incl. gene-start)	ORF	3'UTR(nt) (incl. gene-end)	Intergenic region (nt)	Deduced protein size (aa)
NP	56-1626	72	1398	101	7	465
P (V; W)*	1634-3119	53	1293 (807; 534)	140	2	430 (268; 177)
M	3122-4526	113	1101	191	59	366
F	4586-6420	12	1668	155	49	555
SH	6470-7043	72	429	73	28	142
HN	7072-9102	50	1842	139	63	613
L	9166-16182	112	6726	179		2241

*putative RNA editing site at position 2148-2156 (5'-AAAAAAGGG-3')

**The indicated position is relative to the position of the used reference APMV6/Goose/FarEast/4440/2003 [GenBank:EF569970]

### Phylogenetic analysis based on F and HN proteins

**Figure 1:**
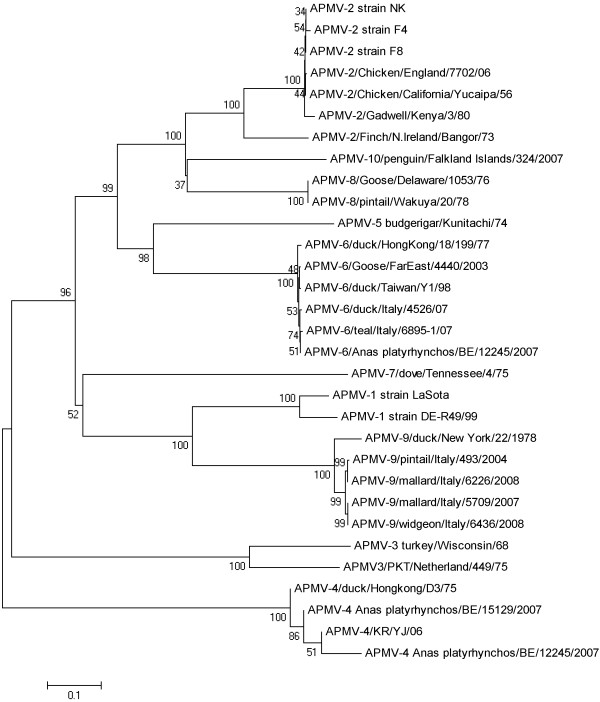
**Phylogenetic tree based on the aa sequences of the F protein**.

**Figure 2:**
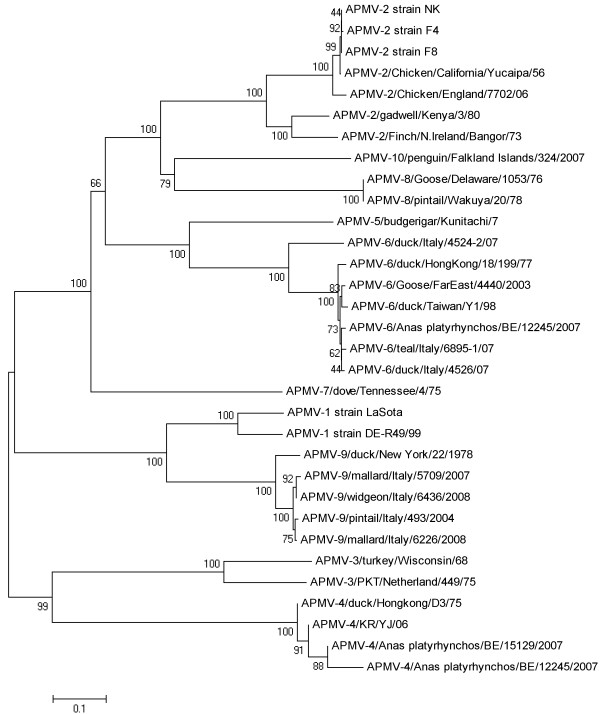
**Phylogenetic tree based on the aa sequences of the HN protein**.

Phylogenetic trees based on amino acid sequence alignments of the F and HN proteins clearly classify APMV4-BE15129 and APMV6-BE12245 within respectively serotype APMV4 and APMV6 (Figure 1 and 2). APMV6-BE12245 is most closely related to the "class I" of APMV6 viruses described by Xiao and colleagues [32]. This is confirmed by its high whole genome nucleotide sequence identity with APMV6/Goose/FarEast/4440/2003 (GenBank:EF569970; Table 4). The F and HN amino acid sequences of APMV4-BE15129 are most closely related to APMV4/KR/YJ/06 (GenBank:EU877976), which is confirmed by a high whole genome nucleotide homology to this virus (Table 5). APMV4-BE15129 is more closely related to both previously sequenced APMV4 whole genomes than these are to each other (Table 5).

**Table 4:** APMV6 complete genomes nucleotide identity matrix

	Hong Kong/18/199/77	Italy/4524-2/07	Taiwan/Y1/98	FarEast/4440/03	APMV-6 BE/12245/07
APMV-6/duck/HongKong/18/199/77	ID				
APMV-6/duck/Italy/4524-2/07	70%	ID			
APMV-6/duck/Taiwan/Y1/98	94.2%	70.2%	ID		
APMV-6/Goose/FarEast/4440/2003	93.8%	70.3%	98.3%	ID	
APMV-6/Anas platyrhynchos/BE/12245/2007	91.9%	69.2%	96%	96.5%	ID

Sites with missing data were deleted from the alignment

**Table 5:** APMV4 complete genomes nucleotide identity matrix

	Hong Kong/D3/75	KR/YJ/06	BE/15129/2007
APMV4/duck/Hong Kong/D3/75	ID		
APMV4/KR/YJ/06	91.6%	ID	
APMV4/Anas platyrhynchos/BE/15129/2007	91.9%	97.6%	ID

Although no complete F and HN sequences were available for APMV4-BE12245 (in sample with APMV6-BE12245), we included the partial sequence information in the phylogenetic analysis using pairwise deletion of positions with gaps and missing data. This may have resulted in biased distance estimations. However, there are clear indications that although it is most closely related to APMV4- BE15129, it is not identical. This is also evident from nucleotide sequence identity calculated over all available sequence information (2977 nt) for the partial genome APMV4-BE12245 (Table 6). The partial sequence APMV4-BE12245 is 98.4% identical to APMV4- BE15129 considering all positions allowed by the partial sequence of APMV-BE12245. In contrast, its identity with previously sequenced APMV4 genomes is only 97.5% (APMV4/KR/YJ/06; GenBank:EU877976) and 90.9% (APMV4/duck/Hong Kong/D3/75; GenBank:FJ177514).

**Table 6:** Nucleotide identity of partial genome sequence (2977nt) of APMV4/mallard/Belgium/12245/07 with APMV4/mallard/Belgium/15129/07 and other APMV4 genomes

	Hong Kong/D3/75	KR/YJ/06	BE/15129/2007	APMV-4 BE/12245/2007
APMV4/duck/Hong Kong/D3/75	ID			
APMV4/KR/YJ/06	91.4%	ID		
APMV4/Anas platyrhynchos/BE/15129/2007	91.3%	97.5%	ID	
APMV4/Anas platyrhynchos/BE/12245/2007	90.9%	97.1%	98.4%	ID

Sites with missing data were deleted from the alignment

## Discussion

Wild birds are increasingly recognized as a reservoir for important livestock diseases. This has been extensively shown for avian influenza A viruses (AIV) and to a lesser degree for avian paramyxoviruses of serotype 1 (APMV1). Moreover, other viruses, including APMV2-10 have been shown to circulate in wild birds. Some of these viruses have been shown to infect poultry species and induced major outbreaks in flocks.

Apart from the well-characterized serotype APMV1 associated with the economically important Newcastle disease in poultry, knowledge of the antigenic and genetic diversity in the APMV serotypes of the genus *Avulavirus *is limited. The determination of complete genome sequences of an additional APMV4 and APMV6 widens our understanding of the genetic diversity in these serotypes. Interestingly, we could identify two different viruses from single pooled samples. In one tested pool of four cloacal swabs, taken in beginning of September, at least one of the four animals was infected with an APMV4. In the other tested pool, taken at the end of this month in the same capture location, two different APMV serotypes APMV6 and APMV4 were identified. The latter APMV4, although closely related to the APMV4 in the first pool, was not identical to it. Contamination artifacts during virus isolation are very unlikely to have occurred as the two APMV4 viruses characterized in this study are not identical based on the sequence information (2977 nt partial sequence of APMV4-BE12245) obtained, and no other APMV4 viruses were manipulated in the laboratory.

It is difficult to assess whether both APMV4 viruses characterized in this study fall within the normal range of quasispecies genetic variation. This is because of the limited availability of sequence information for this serotype and the lack of studies investigating the genetic variability within circulating populations of paramyxoviruses. To prove the economic feasibility of the method of random amplification combined with deep sequencing, the number of sequence reads per sample was intentionally kept below 10 000 in this study. This turned out to be sufficient for the completion of the APMV4 genome in one pool. In the mixed APMV infected pool, this number of reads did not allow the determination of the last 1.11% of the APMV6 genome because part of the sequencing effort resulted in 19.75% of the genome of a co-infecting APMV4. Most probably, the APMV4 virus was present in a lower amount in the original samples, and a higher number of sequence reads would have resulted in completion of the APMV6 genome. However, we cannot fully exclude preferential growth of either virus during virus isolation or a slight bias in our random amplification protocol. This means that quantitative statements about the relative presence of either virus in the original pooled sample based on the distribution of sequence reads are not possible. As the original swabs were no longer available, we could not determine (1) in which proportion the two viruses were present in the original sample/pool before the propagation in eggs, (2) which of the four animals in the pool were infected and (3) whether we were dealing with a mixed infection of one bird. Moreover, the analytical sensitivity of the method remains to be determined and may limit the applicability to field samples containing relatively high virus titers. The presented methodology has the potential to identify viruses present in minor proportions in a pooled sample, and mixed infections in single samples. Clearly our methodology, using a sequence independent methodology for genome determination, has allowed the detection of sequence information from both viruses without bias. In contrast, the use of serotype specific tests such as HI or serotype specific PCR methods may fail to characterize the full complexity of an isolate. Further passage of "double isolates" may give a selective advantage to either virus, changing the biological properties of the isolate, as was suggested by Shihmanter and colleagues [39]. They described that an APMV1 had a selective advantage over co-infecting APMV viruses during passaging in embryonated chicken eggs.

Our genetic identification of the APMVs revealed some difficulties in the HI based identification of APMVs other than APMV1. The APMV6 reference serum did detect the APMV6 virus in sample 07/12245 (titer 1/64) and the APMV4 reference serum detected the APMV4 virus in sample 07/15129 (titer 1/128). However, the HI test failed to detect the APMV4 virus co-present at low titer with the APMV6 virus in pooled sample 07/12245. This most likely indicates that our molecular method is much more sensitive to the identification of viruses present at very low concentrations. Additionally, a cross reactivity with the APMV2 reference serum P/Robin/Hiddensee/57 was observed for both samples (titer 1/32 or 1/64 - Table 1). However another APMV2 reference serum P/chicken/Yucaipa/Cal/56 did not show cross reactivity with these samples, which makes the HI subtyping interpretation difficult. In the context of mixed infections, where it's likely that one virus has a higher concentration than the other, genetic information seems more informative for the identification. Further studies are obviously needed to gain insight in the genetic and antigenic diversity of APMV2-10.

Recently Xiao and colleagues [32] increased the amount of whole genome sequences available for APMV6 to six, identifying two classes with APMV6. APMV6 class I isolates differed less than five % from each other but differed 29-31% to the single class II isolate IT4524-2. The additional APMV6 genome identified in this study clustered within class I, maintaining the separation with class II (31% distance) while slightly increasing the genetic diversity within class I to a maximum of 8% distance.

On the other hand, whole genome sequences of only two representative strains of APMV4 have been reported so far [29,30]. The complete genome of APMV4-BE15129 determined in this study further extends our knowledge of this serotype. This additional APMV4 complete genome does not increase the maximum genetic distance previously documented within the APMV4 serotype. The genetic distance now ranges from two to eight % nucleotide sequence distance (based on only three complete genome sequences). The amount of sequence data compared to APMV1 remains low and further studies are needed to get a better estimate of genetic diversity within serotypes APMV2-10. The sequencing methodology used in this study may facilitate this.

The genome length of 15054 nt for APMV4 and 16236 nt for APMV6 complies with the 'rule of six' for efficient genome replication of *Paramyxovirinae *[40]. The genomic characteristics and genome organizations, including putative mRNA editing of the P gene, are as previously described for APMV4 and APMV6 genomes [29,30,32,33]. Further variability in protein length of the APMV4 M protein was shown. Variability in the intergenic sequence length, as is known for the genus *Avulavirus*, was also confirmed here. A monobasic fusion protein cleavage site was present in both viruses. However, fusion protein cleavage site sequences in APMV2-9 are not necessarily predictive of protease activation phenotype [33], as it is in Newcastle disease virus [41]. Interestingly, the terminal amino acid of the fusion protein cleavage site of APMV4/mallard/Belgium/15129/07 is a phenylalanine. As previously shown for other APMV4 [29,30], this did not require an exogenous exonuclease for in vitro replication on chicken embryonic fibroblasts [29]. A phenylalanine at this position is known to contribute to the in vitro growth characteristics and in vivo pathogenicity of velogenic Newcastle disease [4]. Further in vivo and in vitro phenotypic characterization of this virus would be interesting.

This study clearly demonstrates the value of a sequencing strategy combining next generation sequencing and random access amplification for the identification and whole genome determination of APMVs. Although the method allows sequencing of complete APMV genomes, an unequal distribution of sequencing depth results in low coverage at the genome termini when only a modest sequencing effort is applied. Efforts to optimize the homogenous distribution of sequencing reads along the genome and to determine the optimal sequencing effort for reproducible whole genome sequencing, could further improve the applicability of the method. Previous studies determining complete genomes of APMV2-9 often relied on a round of amplification using degenerated or custom designed oligonucleotides, followed by primer walking [29,31-35]. The use of random access amplification alleviates the problem of oligonucleotide design in a context of poor representation in sequence databases. Moreover, it allows for the identification of potential co-infection with other APMVs or other viruses without methodological bias. Sequence independent single primer amplification (SISPA) was originally described by Reyes and Kim [42]. It was later modified to include enrichment steps for viral nucleic acids using filtration and nuclease treatment (DNase-SISPA, [36,37]). Miller and colleagues [5] used a similar approach for the identification and sequencing of a new serotype of APMV10 in penguins. Unlike their method, that relied on the molecular cloning and sequencing of hundreds of random amplicons, this study used the power of next generation to provide the necessary sequence information. The preparation of a next generation sequencing library includes the process of emulsion PCR, which isolates single DNA molecules on beads and clonally amplifies them ([38], reviewed in [43]). There is no longer a need for molecular cloning and the generated random amplicons can directly be processed in the sequencing library workflow. An additional advantage is that this methodology avoids biological biases induced by the virological analysis of mixed infections.

## Conclusion

Within a single sampling location, three different APMVs were identified in wild mallards using random access amplification in combination with next generation sequencing. From one pooled sample, the complete genome sequence of an APMV4 was assembled from the random sequences. From a second pooled sample, the nearly complete genome sequence of an APMV6 (genome size of 16236 nt) was determined, as well as a partial sequence for an APMV4 closely related but not identical to the APMV4 virus isolated from the first sample.

These data further contribute to the knowledge about the genetic diversity within serotypes APMV4 and APMV6. Moreover, this study demonstrates the value of a random access nucleic acid amplification method in combination with massive parallel sequencing for the characterization and full genome sequencing of APMVs. Moreover, the sequence independent nature of this method allows the detection of potential co-infections with other viruses and is applicable to other viruses.

## Methods

### Viruses

Two non-characterized APMVs (mallard/Belgium/12245/07 and mallard/Belgium/15129/07) were isolated from two pools consisting of each four cloacal swabs from healthy wild mallard ducks according to standard diagnostic procedures (OIE, diagnostic manual 2005/94/CE). The wild birds were caught in a funnel trap located along a pond at 20 km SE of Brussels in Belgium. The trap was visited every two to three days during the entire survey period. All new birds were ringed, weighted, the wings measured, and a cloacal swab was collected. A maximum of four cloacal swabs from the same bird species, sex and sampling time were pooled for laboratory analysis.

### HI-tests

Briefly, the hemagglutination (HA) titer of the different viruses was standardized to a concentration of four units of HA activity/25 μl to perform the test (methodology according to Council Directive 92/66/EC (1992)). All HI tests referenced in this study were conducted with the AIV and APMV1-9 reference sera provided by the European reference laboratory VLA (Weybridge, U.K.). The titer of a serum is defined by the last dilution giving a complete inhibition of HA. A titer below 16 is considered as negative and a titer above or equal to 16 is considered as positive. Absence of APMV1 was confirmed using specific real-time RT-PCR assays (data not shown).

### Random access to viral nucleic acids using DNAse I SISPA

Virus particles from samples mallard/Belgium/12245/07 and mallard/Belgium/15129/07 were purified starting from one ml of allantoic fluid. This was first centrifuged at 3, 200 × g for 15 minutes at four°C to remove cell debris. The supernatants were then filtered at 3, 000 × g for eight minutes or longer at four°C (200 μl/filter) using 0.22 μM filters (Ultrafree-MC GV sterile, Millipore) to remove remaining cell fragments and bacteria. The resulting eluates were subsequently subjected to nuclease treatment with 100 U of DNase I (New England Biolabs) at 37°C for one hour to remove all nucleic acids that are not protected within virions. The resulting virion-enriched samples were used for viral RNA extraction using the QIAamp Viral RNA Mini Kit (Qiagen) according to the manufacturer's instructions.

Sequence independent single primer amplification (SISPA) was performed essentially as previously described [36] with some modifications. Briefly, the extracted RNA was converted into single-stranded cDNA using the Transcriptor First Strand cDNA Synthesis Kit (Roche) and one μM (final concentration) random primer FR26RV-N (5'GCC GGA GCT CTG CAG ATA TCN NNN NN 3', [36,37]). Ten μl extracted RNA was denatured at 95°C for five minutes in the presence of primer FR26RV-N, immediately followed by cooling on ice. The remaining reagents were added. The 20 μl reaction mix contained 1× Transcriptor Reverse Transcriptase Reaction Buffer, dNTP mix (1 mM final concentration each), 20 U Protector RNase Inhibitor, ten units Transcriptor Reverse Transcriptase and one μl PCR-grade H_2_O. The reaction was incubated at 25°C for ten minutes followed by 50°C for 60 minutes. After a reverse transcriptase inactivation step at 85°C for five minutes and chilling on ice, 2.5 U of 3'-5' exo^- ^Klenow Fragment of DNA polymerase (New England Biolabs) were added for second strand synthesis using random primer FR26RV-N for one hour at 37°C. An enzyme inactivation step was performed at 75°C for ten minutes.

Five microliters of the reaction mix was used as template for a subsequent PCR amplification. The 50 μl reaction mix consisted of 1× AmpliTaq Gold^® ^360 DNA buffer, 2.5 mM MgCl2, dNTP mix (0.2 mM final concentration each), 2.5 U AmpliTaq Gold^® ^360 DNA polymerase (Applied Biosystems), 32.7 μl RNase free water and 1.6 μM FR20RV primer (5'-GCC GGA GCT CTG CAG ATA TC-3', [36,37]). This PCR primer is complementary to the amplification tag of FR26RV. The reaction was incubated at 95°C for ten minutes, 40 cycles at 95°C for one minute, 48°C for one minute and 72°C for two minutes followed by a final elongation for seven minutes at 72°C.

The random amplified DNA fragments were visualised on a one % agarose gel. Fragments of 400-1000 base pairs (bp) were excised and purified from the gel with the High Pure PCR Product Purification Kit (Roche). The purified PCR fragments were quantified by spectrophotometry (Nanodrop-1000).

### Sequencing

Five micrograms of size selected (400-1000 bp) purified random amplified DNA was sequenced on a GS FLX (Roche, Mannheim, Germany) by the Genomics Core of the University Hospital, University of Leuven, Belgium. They used multiplex identifier (MID) identification during library preparation (standard Roche MID tag sequences) and GS FLX Titanium series reagents (Roche, Mannheim, Germany) according to their standard procedures, aiming for 5000-10000 reads per library. Briefly, adaptors including standard MID tag sequences (for our samples RL3 and RL10) were ligated to the size selected double stranded DNA library (Rapid Library Preparation Method Manual, GS FLX Titanium Series reagents, Roche, Mannheim, Germany), followed by single stranded DNA library isolation and library quality assessment and quantitation. The resulting libraries were then pooled with other MID identified libraries and emulsion PCR clonal amplification was performed as described by the provider. The amplified libraries were then loaded on a Pico Titer Plate for sequencing by the Genome Sequencer FLX. Data were provided to the authors by secured ftp-server.

### DATA Analysis

The obtained raw sequence data were assembled using SeqMan NGen^® ^version 3.0 (DNASTAR, Madison, WI, USA). The reads were trimmed to remove primer sequences as well as low quality ends. Standard assembling and filtering parameters were used. First we performed a de novo assembly and entered the resulting contigs (i.e. sets of overlapping sequence reads) into a *Blastn *similarity search against public sequence databases (http://blast.ncbi.nlm.nih.gov/Blast.cgi; [44]) for identification. When we identified a certain APMV serotype, we used the blast hit with the highest identity of the biggest APMV contig as reference genome for a subsequent reference assembly with the same raw data set. The resulting reference assembly was used to obtain a complete genome consensus sequence. The sequence reads contributing to the consensus were also checked for variability. When at a certain position along the consensus two different nucleotides were present, the variability was indicated as an ambiguous nucleotide when the minor nucleotide exceeded the threshold of one third of the reads.

### Analysis of the virus specificity of the protocol

Sequences failing to align with the used reference genome were subjected to a metagenomics assembly in SeqMan NGen. The obtained contigs containing more than two sequence reads were identified with *megablast *http://blast.ncbi.nlm.nih.gov/Blast.cgi. Sequences were classified as previously described [36]. Briefly, viral blast results were considered reliable if the best hit had an E-value less than 10^-25^. Non-viral sequences were identified as *Gallus gallus *(chicken embryos were used for virus isolation), other birds, bacteria, ... if their best hit was below an E-value of 10^-10^. If no blast results were found or the E-value was below the 10^-10 ^cut off value, the sequences were not given a specific designation.

### Phylogenetic analysis

Consensus sequences were edited, aligned and translated, and sequence identities were calculated using Bioedit v 7.0.5.3 http://www.mbio.ncsu.edu/bioedit/bioedit.html[45]. Nucleotide (nt) sequence identities with selected complete genome sequences were determined (GenBank accession codes: EU877976, FJ177514, EF569970, NC003043, GQ406232, EU622637). Amino acid (aa) alignments (ClustalW algorithm) using all available complete coding sequences for the F and HN genes of APMV4 (GenBank accession codes FJ177514, EU877976) and APMV6 (GenBank accession codes EF569970, NC003043, EU622637, GQ406234, GQ406233) and selected sequences representative of other APMV serotypes (GenBank accession codes AF077761, DQ097393, HQ896024, HQ896023, HM159993- HM159995, AY129676, EU338414, EU782025, EU403085, GU206351, FJ231524, FJ619036, FJ215864, GU068584-GU068587, EU910942, HM147142) were used for phylogenetic analysis. Mega v5.01 [46] was used to construct phylogenetic trees by bootstrap analysis (1000 replicates) using the neighbour-joining of the Poisson-corrected values for aa differences. All positions containing alignment gaps and missing data were eliminated only in pairwise sequence comparisons, allowing the inclusion of the incomplete aa F and HN sequences of APMV4/mallard/Belgium/12245/07 (APMV4-BE12245) in the analysis.

## List of abbreviations used

aa: amino acids; AIV: avian influenza A virus; APMV: avian paramyxovirus; bp: base pairs; F: fusion protein; HA: hemagglutination; HI: hemagglutination inhibition; HN: hemagglutinin-neuraminidase; M: matrix protein; MID: multiplex identifier; L: large polymerase; NDV: Newcastle disease virus; NP: nucleoprotein; nt: nucleotides; ORF: open reading frame; P: phosphoprotein; SH: small hydrophobic protein; SISPA: sequence independent single primer amplification.

## Competing interests

The authors declare that they have no competing interests.

## Authors' contributions

TR performed the molecular biology and sequence analysis work and contributed to the manuscript draft. BL provided the samples and realized the virological characterization. FV and TV contributed to the interpretation of the data. SV conceived and supervised the study, realized the phylogenetic and genomic analysis, and contributed to the manuscript draft. All authors read and approved the final manuscript.
